# Application of Suspension Fixation with Button Plates for Patients with Distal Radioulnar Joint Dislocation: A Case Series

**DOI:** 10.1111/os.12932

**Published:** 2021-10-01

**Authors:** Hongliang Liu, Shuchai Xu, Zexin Huang, Yang Lv, Bojian Chen, Xiaodong Lin, Jun Liu, Lili Sang

**Affiliations:** ^1^ Department of Orthopaedics The Second Affiliated Hospital of Guangzhou University of Traditional Chinese Medicine Guangzhou China; ^2^ Division of Joint Surgery, Department of Orthopaedic Surgery The Affiliated Zhongshan Hospital of Guangzhou University of Traditional Chinese Medicine Zhongshan China

**Keywords:** Dislocation, New application, Subluxation

## Abstract

**Objective:**

The aim of the present study was to assess the effect of suspension fixation with button plates on the reconstruction of the distal radioulnar joint dislocation (DRUJ).

**Methods:**

This was a case series of six patients (two men and four women) who underwent suspension fixation with button plates for DRUJ dislocation between January 2015 and May 2017. Physical examination, radiography, MRI, functional activity of the wrist joint, grip strength of the wrist joint, Garland–Werley wrist score, Mayo wrist score, and visual analog scale (VAS) score were used to evaluate the effect of this procedure. All patients were followed up every 3 months. The evaluation time point was 12 months after the operation. Comparisons of the functional indexes of wrist function before and after the operation were performed using paired statistical tests.

**Results:**

The mean range of motion of the affected limb was 70° at forearm pronation and 75° at forearm supination. The subjective assessments and tests of the motor function of the wrist showed improvement after surgery. The Garland–Werley wrist score was 13.50 ± 2.66 preoperatively, the Mayo wrist score was 56.67 ± 18.35, and the VAS score was 4.83 ± 1.17. The Garland–Werley wrist score was 2.83 ± 1.33 postoperatively at 12 months, the Mayo wrist score was 87.5 ± 6.89, and the VAS score was 0.50 ± 0.55. At 12 months, the Garland–Werley wrist score, the Mayo wrist score, and the VAS score showed significant improvements when compared with those before surgery (*P* = 0.000, *P* = 0.003, and *P* = 0.000, respectively). Radiographic examination revealed that the internal fixation device was in place, and no dislocation of the DRUJ could be observed. None of the patients had internal fixation device removal or re‐dislocation of the DRUJ. None of the patients had re‐dislocation of the DRUJ. No secondary ulnar or radial fractures and nerve injury were reported during and after surgery. No tumor recurrence was observed in patients with giant cell tumors of the tendon sheath. No loosening and displacement of screws were reported.

**Conclusion:**

The new method of suspension fixation with button plates for the surgical reconstruction of a DRUJ dislocation is simple, with minimal trauma, and maintains the stability of the DRUJ without the need for intra‐articular or extra‐articular reconstruction of the ligament. Furthermore, it allows early functional exercise and achieves satisfactory postoperative functional recovery.

## Graphical abstract

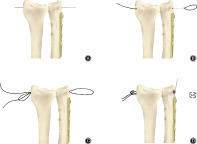

The new method of suspension fixation with button plates for the surgical reconstruction of a DRUJ dislocation is simple, with minimal trauma, and maintains the stability of the DRUJ without the need for intra‐articular or extra‐articular reconstruction of the ligament. The process of the new application of suspension fixation for the DRUJ reconstruction: (A) A Kirschner wire was used to drill a tunnel into the DRUJ. (B) and (C) The suture was pulled tight and knotted. (D) Addition of the button plates to fix the DRUJ.

## Introduction

Distal radioulnar joint (DRUJ) dislocation is often associated with distal ulnar and radius fractures, accounting for approximately 10%–19% of distal ulnar and radius fractures^1^. The DRUJ consists of an annular articular surface on the ulnar head and an ulnar notch of the radius^2^. The distal edge of the notch has a triangular fibrocartilaginous disc attached to the base of the ulna styloid process^2^. The stability of the DRUJ is mainly maintained by the bony structure of the joint and the surrounding soft tissues, including the triangular fibrocartilage complex (TFCC), the distal interosseous membrane (DIOM), and the pronator quadratus (PQ)^2^. Among them, the TFCC is the most important structure for maintaining the stability of the DRUJ. The TFCC mainly consists of the distal ulnar ligament, the triangular fibrocartilage disc, the meniscus, and the ulnar extensor tendon of the wrist. The bony structures provide only approximately 20% of the stability^3^. The DRUJ is regarded as the most important anatomical structure for maintaining the normal rotation of the forearm^4^. Dislocation of the DRUJ might seriously affect the functioning of the forearm and wrist joint^2^.

Nevertheless, no uniform treatment is presently recognized for DRUJ dislocation^5^, ^6^, ^7^, ^8^, ^9^, ^10^, ^11^, ^12^. The anatomical reconstruction of the distal ulnar radial ligament and extraarticular fixation of the distal ulnar radial ligament are the most widely practiced approaches for DRUJ dislocation^13^, ^14^. Among them, the former represents the Adams–Berger procedure^15^. This is a complex technique that requires cutting of the articular capsule, and this, in turn, might damage important anatomical structures such as the triangular fibrocartilage complex, affecting recovery^16^. The latter technique involves internal fixation with a Kirschner wire but there are potential complications, such as secondary fractures, Kirschner wire breakage, and joint stiffness^13^, ^14^. The Sauve–Kapandji procedure is the gold standard and involves distal ulnar excision and radioulnar joint fusion^17^. This approach improves the rotation function of the distal radioulnar joint and relieves pain, correcting the impact of the ulnar region, but it is not conducive to the stability of the DRUJ^18^. Although these reconstruction methods are applied clinically, they have their own shortcomings.

The injury characteristics of the DRUJ were collected and summarized, and were reported through a series of cases. This study: (i) describes a new method for the surgical reconstruction of the DRUJ using suspension fixation with a button plate; (ii) provides conditions for early functional rehabilitation exercise, avoiding a series of complications such as internal fixation failure and joint stiffness^19^; and (iii) assesses the safety and indications of suspension fixation with button plates in the reconstruction of the DRUJ based on physical examination, radiography, MRI, functional activity of the wrist joint, grip strength of the wrist joint, Garland–Werley wrist score, Mayo wrist score, and visual analog scale (VAS) score.

## Methods

### Diagnositc Criteria, Inclusion Criteria, and Exclusion Criteria

#### Diagnostic Criteria

Patients in the study had acute or chronic DRUJ dislocations, including traumatic DRUJ dislocations or Colles fractures, Smith fractures, Galazzi fractures combined with DRUJ dislocation, chronic inflammation involving the DRUJ, and tumors involving the DRUJ. The symptoms and signs of DRUJ dislocation included positive “ulnar fossa sign,” “piano key sign,” and positive forearm rotation test^1^, ^2^, ^15^. The diagnosis of DRUJ was made by combining the positive and lateral X‐ray films. The contralateral X‐ray films should be added if necessary. CT or MRI could be used to confirm the diagnosis^15^.

#### Inclusion Criteria

The inclusion criteria were: (i) patients 18 to 75 years old who were diagnosed with DRUJ dislocation; (ii) patients who underwent DRUJ construction; and (iii) all the follow‐up data for physical examination, radiography, MRI, functional activity of the wrist joint, grip strength of the wrist joint, Garland–Werley wrist score, Mayo wrist score, and VAS score before and 12 months after the operation had been collected.

#### Exclusion Criteria

The exclusion criteria were: (i) patients who disagreed with the surgical plan, were unwilling to participate in experimental research, or did not cooperate with the treatment; (ii) patients with complications such as serious cardiovascular and cerebrovascular diseases, and liver, kidney or hematopoietic system diseases; (iii) patients with multiple trauma (injury severity score [ISS] >16), with fever or skin allergies, with mental illness or Alzheimer's disease or with severe open fractures, and pregnant women were excluded.

### General Data

From January 2015 to May 2017, six patients diagnosed with DRUJ dislocation by radiography, MRI scan, and physical examination were admitted to our hospital. This study was approved by the Ethics Committee of our hospital (B2017‐153). The patients included two men and four women, with a mean age of 41.5 ± 14.2 years (ranging from 26 to 60 years old). The mean medical history of the patients was 17.0 ± 14.9 months (range, 1–36 months). Follow‐up was performed in all six patients. The mean follow‐up was 26.5 ± 9.4 months (range, 15–42 months) (Table 1).

**TABLE 1:** General data of the patients

Number	Sex	Age (years)	Disease course (months)	Follow‐up period (months)	Accompanying conditions	Surgical plan
1	F	50	24	42	Giant cell tumor of the tendon sheath and a long ulna	Tumor removal + ulnar shortening osteotomy + suspension fixation
2	F	31	30	27	Long ulna	Ulnar shortening osteotomy and internal fixation + suspension internal fixation
3	M	26	8	30	Radioulnar fractures	Open reduction and internal fixation + suspension fixation
4	M	52	3	19	Radioulnar fractures	Open reduction and internal fixation + suspension fixation
5	F	60	36	15	Long ulna	Ulnar shortening osteotomy and internal fixation + suspension internal fixation
6	F	30	1	26	None	Suspension internal fixation
Range	/	26–60	1–36	15–42	/	/
Mean	/	41.50 ± 14.20	17.00 ± 14.91	26.50 ± 9.40	/	/

General data of the patients are shown in Table 1. The measurement data is expressed as Mean±SD. F, female; M, male; /, no need to fill in.

### Treatment

#### Management of Primary Disease

**Fig. 1:**
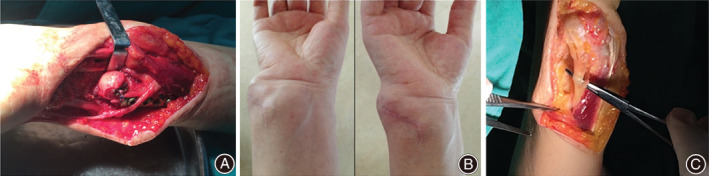
A patient with a tendon sheath tumor who underwent distal radioulnar joint (DRUJ) reconstruction. (A) Preoperative appearance of the tumor. (B) Appearance of the DRUJ after removing the tumor. (C) Appearance of the wrist before and after surgery.

All procedures were performed by the same experienced chief physician. The patients were placed in the supine position. After the successful induction of brachial plexus anesthesia, the operative field of the upper arm was routinely disinfected and draped. A tourniquet was placed on the arm proximal to the operation field. The management of one patient was complicated: the patient had multiple giant cell tumors of the tendon sheath invading the DRUJ, as well as a long ulna. The tumors were removed, and ulnar osteotomy and plate and internal screw fixation were performed (Fig. 1). Two patients with long ulnas underwent ulnar osteotomy and plate and internal screw fixation. Three patients with traumatic dislocation of the DRUJ underwent internal fixation with plates and screws. After completing the treatment of the primary diseases, the DRUJ was reconstructed by suspension fixation with button plates.

#### Suspension Fixation with Button Plate

**Fig. 2:**
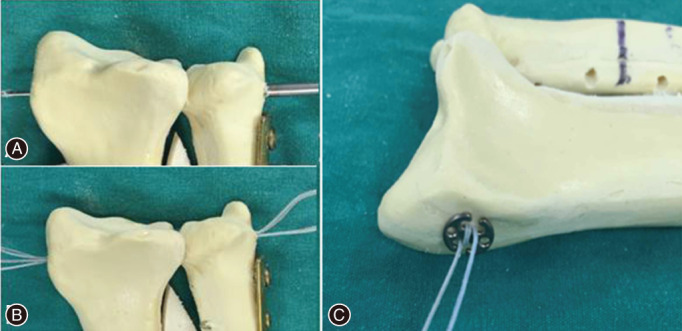
The process of the new application of suspension fixation for the distal radioulnar joint (DRUJ) reconstruction. (A) A Kirschner wire was used to drill a tunnel into the DRUJ. (B) Two 6‐mm (in diameter) button plates were attached to the radial or ulnar side of the DRUJ by a super‐suture. (C) The suture was pulled tight and knotted.

**Fig. 3:**
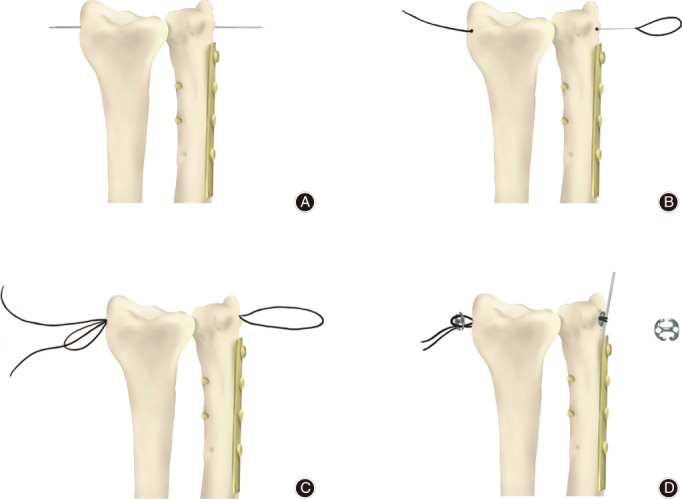
The drawing of distal radioulnar joint (DRUJ) reconstruction. (A) A Kirschner wire was used to drill a tunnel into the distal radioulnar joint (DRUJ). (B and C) The suture was pulled tight and knotted. (D) The button plates are added to fix the DRUJ.

Distal radioulnar joint dislocation reconstruction was performed after completion of the primary surgery. A 2‐mm Kirschner wire (AO Company, Davos, Switzerland) was used to drill a tunnel into the DRUJ from the radius to the ulna. Next, two 6‐mm (in diameter) button plates were attached to the radius or ulnar side of the DRUJ by a super‐suture (Smith‐nephew & Johnson Medical Device Company, USA), forming four strands of thread by going through the button plates twice using a special stringing tool. The sutures were tightly pulled to make the ulnar articular surface (radius side) attach to the sigmoid notch of the distal radius. A knot was then formed to complete the fixation. The schematic diagram of this approach is shown in Fig. 2. At this point, the “piano key sign” and the sign of “ulnar fovea sign” became negative. The DRUJ remained stable during forearm rotation. The surgical wound was rinsed and closed in layers. A drawing of the technique is provided in Fig. 3.

### Rehabilitation After Surgery

The surgical site was dressed using cotton pads with pressure to maintain the functional position of the right wrist. On postoperative day 1, the non‐weight‐bearing exercise of the forearm was initiated, which included rotation, flexion, and extension exercises. Two patients with fractures of the ulnar and radius were treated with a plaster cast for 3 weeks. Rehabilitation exercises were started after cast removal.

### Follow‐Up and Evaluation

All patients were followed up every 3 months. The last follow‐up was at 42 months. Physical examination, Garland–Werley wrist score, Mayo wrist score, and VAS score were used to evaluate the effect of this procedure. Radiography and MRI were performed to verify the imaging diagnosis of DRUJ construction. The evaluation time point was 12 months after the operation.

### Outcome Measures

#### Functional Activity of the Wrist Joint

The functional activities of the wrist joint assessed before and 12 months after the operation include pronation, supination, flexion, extension, ulnarduction, and radialduction.

#### Grip Strength of the Wrist Joint

The grip strength of the wrist joint was evaluated before and 12 months after the operation. Preoperative grip strength and postoperative grip strength were calculated by comparison of the affected and the contralateral wrist joint.

The Garland–Werley wrist score, the Mayo wrist score, and the VAS score were used to evaluate the effect of this procedure before and 12 months after the operation.

1. The Garland–Werley wrist score is used to assess the residual deformity and complications and for subjective and objective evaluation of the wrist. In the evaluation, “0–2” is regarded as “excellent,” “3–8” as “good,” “9–20” as “ fair,” and “≥21” as “poor.”
2. The Mayo wrist score system is used to evaluate the level of disability in the wrist, and to assess pain, functional status (able to work), range of motion, and grip strength. The Mayo wrist score assesses four domains: pain, grip strength, range of motion, and return to employment. Each domain is scored out of 25 points to produce a total score out of 100 points. The Mayo wrist score ranges from 0 to 100, with a score of 0 indicating a worse wrist condition and 100 indicating a better wrist condition.
3. The VAS is a simple method for measuring pain intensity in clinical practice. The basic method is to use a scale with a length of approximately 10 cm, with one side marked with 10 lines. The score is determined by measuring the distance between the two ends, with “0” and “10” at each end; “0” represents no pain and “10” represents the most severe pain that is unbearable. In clinical use, the side with the scale should be turned back to the patient, and the patient marks the corresponding position on the ruler that represents their degree of pain.

### Statistical Analysis

Statistical analysis was performed using SPSS 22.0 (IBM, Armonk, NY, USA). All measurement data (age, disease course, follow‐up period, functional activity of the wrist joint, and functional scoring of the wrist joint) (mean ± standard deviation) were examined initially using repeated measure ANOVA, the paired *t*‐test, or the Wilcoxon test. Categorical data (comparison of affected and contralateral grip strength of the wrist joint) were presented as numbers and percentages and were analyzed using the McNemar test. The statistical difference level was set at <0.05.

## Results

### General Results

No secondary ulnar or radial fractures and nerve injury were reported during and after surgery. No tumor recurrence was observed in patients with giant cell tumors of the tendon sheath. Two patients with long ulnas underwent an osteotomy. During follow up, no loosening and displacement of screws were reported.

### Intraoperative Results

During the operation, the forearm was rotated, and the range of motion could reach 90° of pronation and supination. There was no obvious loosening of internal fixation during the activity, and the piano key sign was negative.

### Outcome Measures

Functional activity of the wrist joint before and 12 months after the operation is shown in Table 2. The preoperative degrees of pronation, supination, flexion, extension, ulnarduction, and radialduction were 71.67° ± 19.15°, 61.67° ± 22.95°, 60.83° ± 26.16°, 60.0° ± 30.17°, 8.33° ± 4.82°, and 14.17° ± 7.36°, respectively. The degrees of pronation, supination, flexion, extension, ulnarduction, and radialduction at 12 months after surgery were 80.83° ± 11.14°, 75.00° ± 14.83°, 74.17° ± 20.84°, 71.67° ± 21.13°, 16.67° ± 2.58°, and 16.67° ± 4.08°, respectively.

**TABLE 2:** Functional activity of the wrist joint before and 12 months after the operation

Number	Pronation (degree)		Supination (degree)		Flexion (degree)		Extension (degree)		Ulnarduction (degree)		Radialduction (degree)	
	Before	After	Before	After	Before	After	Before	After	Before	After	Before	After
1	90	90	70	80	70	90	75	85	15	20	20	20
2	90	90	90	90	90	90	90	90	5	15	20	20
3	45	65	40	60	30	45	30	50	5	15	5	10
4	60	70	45	55	30	50	15	40	10	15	5	15
5	85	90	85	90	85	85	80	80	5	15	20	20
6	60	80	40	75	60	85	70	85	10	20	15	15
Mean ± SD	71.67 ± 19.15	80.83 ± 11.14	61.67 ± 22.95	75.0 ± 14.83	60.83 ± 26.16	74.17 ± 20.84	60.0 ± 30.17	71.67 ± 21.13	8.33 ± 4.82	16.67 ± 2.58	14.17 ± 7.36	16.67 ± 4.08

General functional activity of the wrist joint before and 12 months after the operation is shown. The measurement data is expressed as Mean ± SD.

Comparison of affected and contralateral grip strength of the wrist joint is shown in Table 3. The average grip strength (affected/contralateral) before and 12 months after the operation were 72% and 84%, respectively. At 12 months after surgery, the subjective assessments and tests of the motor function of the wrist demonstrated improvement when compared with those before surgery.

**TABLE 3:** Comparison of affected and contralateral grip strength of wrist joint

Number	Preoperative grip strength, affected/contralateral (%)	Postoperative grip strength, affected/contralateral (%)
1	80	95
2	95	93
3	36	58
4	64	72
5	88	94
6	70	93
Mean	72	84

Comparison of affected and contralateral grip strength of the wrist joint before and 12 months after the operation.

Functional scoring of the wrist joint before and 12 months after the operation is shown in Table 4. The Garland–Werley wrist score was 13.50 ± 2.66 preoperatively, the Mayo wrist score was 56.67 ± 18.35, and the VAS score was 4.83 ± 1.17. The Garland–Werley wrist score was 2.83 ± 1.33 postoperatively at 12 months after surgery, the Mayo wrist score was 87.5 ± 6.89, and the VAS score was 0.50 ± 0.55. At 12 months after surgery, the Garland–Werley wrist score, the Mayo wrist score, and the VAS score showed significant improvements when compared with those before surgery (*P* = 0.000, *P* = 0.003, and *P* = 0.000, respectively).

**TABLE 4:** Functional scoring of wrist joint before and 12 months after the operation

Number	Garland–Werley wrist score		Mayo wrist score		Visual analog scale	
	Before	After	Before	After	Before	After
1	16	3	65	90	5	1
2	10	2	70	95	4	0
3	17	5	20	75	7	1
4	12	3	65	85	4	0
5	14	3	60	90	5	1
6	12	1	60	90	4	0
*u‐*value	8.77		−3.85		8.22	
*P*‐value	<0.001*		<0.01*		<0.001*	

Functional scoring of wrist joint before and 12 months after the operation.

^†^ Statistical difference between the general functional activity of the wrist joint before and 12 months after operation level was set at <0.05.

* Statistically significant difference was set at <0.01.

### Stability of the Internal Fixation Device

**Fig. 4:**
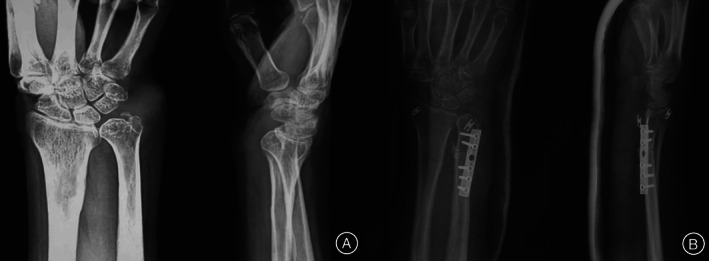
A patient with the new application of suspension fixation for the distal radioulnar joint (DRUJ) reconstruction. (A) X‐ray films show multiple giant cell tumors of the tendon sheath invading the DRUJ and leading to ulna impaction. (B) Postoperative X‐ray films of the patient who underwent tumor removal, osteotomy (8‐mm long excision of the ulna), internal fixation with plate and screws, and suspension fixation of the DRUJ.

**Fig. 5:**
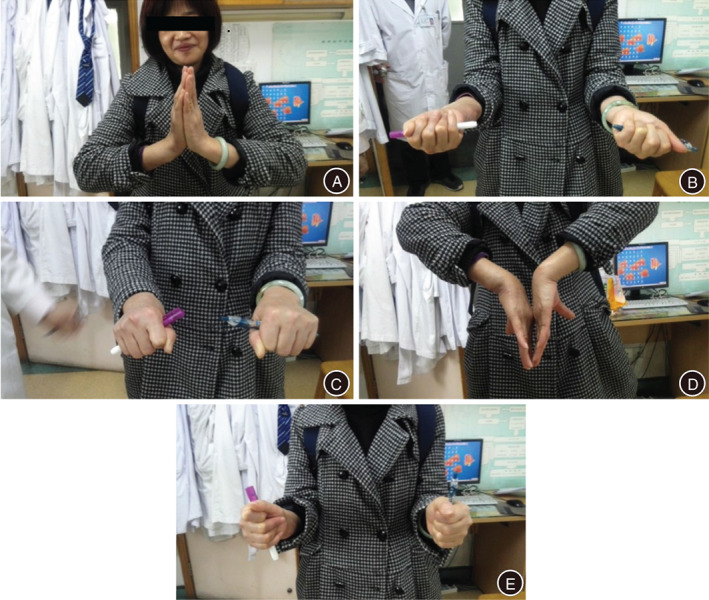
The right arm positions after the distal radioulnar joint (DRUJ) reconstruction of the patient with multiple giant cell tumors. (A) Dorsiflexion (right 85°, left 90°). (B) Supination (right 80°, left 90°). (C) Pronation (right 90°, left 90°). (D) Palmar flexion (right 90°, left 90°). (E) Neutral position.

At the final follow‐up, radiographic examination revealed that the internal fixation devices were in place. Repeated radiography and MRI showed stable DRUJ and good arrangement of the joint. No apparent dislocation and disordered arrangement between the distal radius and the ulna were observed (Fig. 4). No obvious dislocation of DRUJ occurred due to early internal fixation loosening. None of the six patients had an internal fixation device removed. Physical examination at the final follow‐up demonstrated that the “piano key sign” and the “ulnar fovea sign” were negative (Fig. 5).

### Complications

Two patients had ulna and radius fractures. One patient had multiple giant cell tumors of the tendon sheath, and three patients had a long ulna. During follow‐up, none of the patients had re‐dislocation of the DRUJ. No secondary ulnar or radial fractures and nerve injury were reported during and after surgery. No tumor recurrence was observed in patients with giant cell tumors of the tendon sheath. Two patients with long ulnas underwent an osteotomy. During follow up, no loosening and displacement of screws were reported.

## Discussion

Distal radioulnar joint dislocation involves traumatic and pathological dislocation. Traumatic or chronic inflammation affects the bony structure and soft tissues of the DRUJ, leading to instability or dislocation. Typical signs of DRUJ dislocation include the “ulnar fovea sign,” the “piano key sign”, the “positive forearm rotation test,” the “positive squeeze test,” and ulnar impact syndrome^1^, ^2^, ^15^. Studies reported that pain in the ulnar fovea site is a sensitive sign of TFCC injury (sensitivity: 95.2%)^20^. Biomechanical studies have confirmed that ulnar impact syndrome is associated with DRUJ instability^21^. Positive physical signs and anteroposterior views of radiographs were used for diagnosis and, if necessary, a contralateral wrist radiograph was obtained for comparison^22^. CT or MRI can also be used for diagnosing DRUJ^23^. In addition, wrist arthroscopy is an effective surgical treatment option, and is also the gold standard for DRUJ diagnosis under direct vision, especially for TFCC injury^24^.

The instability of acute DRUJ can be treated by external fixation with plaster cast and by repairing the TFCC. Inappropriate treatment of the DRUJ can cause wrist pain, limiting the rotation of the forearm. Successful conservative treatment has been reported previously. Wassink achieved favorable treatment results by manual reduction and plaster cast fixation in a patient with repeated DRUJ dislocation with trauma^25^.

Soft tissue reconstruction is the first choice of treatment for chronically unstable or dislocated DRUJ that has not yet degenerated. Biomechanical studies showed that there are some differences in the physiological status of the trajectories of articular motion after reconstruction, except for articular capsule shortening^14^. Ulnar tendon suspension fixation induces poor stability^26^ and has been mainly used for treating ulnar instability. Although the shortening of the extensor support band and joint capsule have minimal effects on DRUJ motion trajectories, they are currently used for restoring mild instabilities or used in combination with other soft tissue reconstructions. According to a retrospective study by El‐Haj *et al*. (2017)^27^, satisfactory results were obtained during short‐term follow‐up (16 months) for treating dorsal instability of the DRUJ by shortening the dorsal wrist support band.

The representative procedure for DRUJ ligament reconstruction is the Adams and Berger procedure^15^, but the operation is complicated. This procedure requires the incision of the support band as well as the joint capsule for intra‐capsule operation. A tunnel has to be drilled in the ulnar joint fossa to allow the tendon to travel through it. This technique might impact the blood supply of the TFCC and might even directly damage the anatomy of the TFCC^16^. Thus, both the postoperative bone and the joint tissue repair can be compromised. In addition, this type of anatomic reconstruction cannot truly simulate the anatomical and biomechanical properties of the deep and superficial layer structures of the DRUJ ligament. These factors might impact surgical outcomes and increase the incidence of complications. In this study, the suspension fixation method with a button plate to reconstruct the DRUJ was found to be simple for the surgeon to study. It can also support early functional exercise and can achieve satisfactory postoperative functional recovery.

Extra‐articular fixation of the DRUJ (e.g. Kirschner wire fixation of the DRUJ), which has less impact on TFCC, cannot meet the needs for postoperative rehabilitation exercises and can easily cause postoperative forearm stiffness. This procedure is associated with complications, including Kirschner wire breakage and secondary fractures.

The suspension manipulation technique is minimally invasive and can be easily performed. Even if the fixation fails, an alternative is still available. Drake^28^ and Kam *et al*.^29^ used a suture button device for reconstructing the stability of the DRUJ. For patients with DRUJ instability, de Vries^30^ performed biomechanical experiments to demonstrate that the suture button device can be used to reconstruct the distal interosseous membrane for treating instability of the DRUJ and for reconstructing DRUJ stability. In patients with DRUJ instability and with dislocation caused by TFCC injury, Shuchai Xu, an author of the present study, adopted an improved suspension device for reconstructing DRUJ stability and achieved satisfactory clinical efficacy^19^. Xu's procedure is simple, minimally traumatic, reproducible, and beneficial for early postoperative rehabilitation, which subsequently presents new options for reconstruction of DRUJ stability in the future, without the need for intra‐articular or extra‐articular reconstruction of the ligament. The procedure does not damage the adjacent structures of the joint and can support early functional exercise and achieve satisfactory postoperative functional recovery.

Drilling a tunnel with a 2‐mm Kirschner wire reduces the diameter of the bone tunnel, which, in turn, reduces the risk of fractures due to an oversized tunnel during ligament reconstruction. Meanwhile, the procedure can reduce the regional injury of the TFCC attachment site and can provide a stable environment for good healing. Compared with a free tendon transplantation procedure, this procedure has the smallest effect on the donor site and can maintain the vitality and tension of the tendon. Especially for patients with DRUJ instability or dislocation and severe traumatic injury of the donor ligament due to pathological factors, the suspension technique easily achieves stable fixation and also greatly reduces the surgical trauma. In addition, the suspension fixation allows patients to start early forearm rotation exercises and avoids internal fixation fractures or joint stiffness due to a rigid fixation (i.e. Kirschner wire fixation).

Our previous study of eight cadaveric specimens showed that the biomechanical performance of using the button plate suspension fixation to reconstruct the DRUJ and ulnar joint could meet the requirements for the stable reconstruction of the DRUJ and ulnar joint, and provided data supporting the use of this technique in the clinic^31^. The biomechanical tests showed that the torque of the forearm pronation/supination within 45° was an acceptable range, and it would not cause failure of the fixation. Therefore, this procedure allows patients to perform functional exercises with pronation/supination within 45° at an early stage, which can effectively prevent soft tissue adhesion.

Our study has limitations. This is a descriptive and retrospective analysis, with the inclusion of a small sample size due to this being a single‐center study. The patients had various causes of DRUJ dislocation, possibly introducing some bias in the outcomes. In addition, the study lacked a control group, and the follow‐up time was limited.

This study describes a new method for the surgical reconstruction of the DRUJ through suspension fixation with a button plate. Postoperative follow up showed a good position of the DRUJ with no significant dislocation or subluxation. This procedure is simple and less traumatic for the reconstruction of DRUJ stability, without the need to perform intra‐articular or extra‐articular reconstruction of the ligament. This procedure does not damage the adjacent structures of the joint and can support early functional exercise and achieve satisfactory postoperative functional recovery. Thus, the suspension fixation technique is an effective and novel approach for DRUJ reconstruction.
